# Fish Product Mislabelling: Failings of Traceability in the Production Chain and Implications for Illegal, Unreported and Unregulated (IUU) Fishing

**DOI:** 10.1371/journal.pone.0098691

**Published:** 2014-06-12

**Authors:** Sarah J. Helyar, Hywel ap D. Lloyd, Mark de Bruyn, Jonathan Leake, Niall Bennett, Gary R. Carvalho

**Affiliations:** 1 Molecular Ecology and Fisheries Genetics Laboratory, Bangor University, Bangor, Wales, United Kingdom; 2 Sunday Times, London, United Kingdom; 3 Greenpeace UK, London, United Kingdom; Aristotle University of Thessaloniki, Greece

## Abstract

Increasing consumer demand for seafood, combined with concern over the health of our oceans, has led to many initiatives aimed at tackling destructive fishing practices and promoting the sustainability of fisheries. An important global threat to sustainable fisheries is Illegal, Unreported and Unregulated (IUU) fishing, and there is now an increased emphasis on the use of trade measures to prevent IUU-sourced fish and fish products from entering the international market. Initiatives encompass new legislation in the European Union requiring the inclusion of species names on catch labels throughout the distribution chain. Such certification measures do not, however, guarantee accuracy of species designation. Using two DNA-based methods to compare species descriptions with molecular ID, we examined 386 samples of white fish, or products labelled as primarily containing white fish, from major UK supermarket chains. Species specific real-time PCR probes were used for cod (*Gadus morhua*) and haddock (*Melanogrammus aeglefinus*) to provide a highly sensitive and species-specific test for the major species of white fish sold in the UK. Additionally, fish-specific primers were used to sequence the forensically validated barcoding gene, mitochondrial cytochrome oxidase I (COI). Overall levels of congruence between product label and genetic species identification were high, with 94.34% of samples correctly labelled, though a significant proportion in terms of potential volume, were mislabelled. Substitution was usually for a cheaper alternative and, in one case, extended to a tropical species. To our knowledge, this is the first published study encompassing a large-scale assessment of UK retailers, and if representative, indicates a potentially significant incidence of incorrect product designation.

## Introduction

In recent years, concerns about the health of the oceans and the effects of over-exploitation of fisheries have increased. Consumer demand for seafood is growing with the contribution of fish to the average annual diet reaching a record of 18.8 kg per person per year in 2011 [1], as compared to 17.1 Kg in 2008 [2]. This is partly due to an increase in the range of species consumed, and an increase in aquaculture. Fish products were worth a record $217.5 billion in 2010, up over 9% from 2009, and these trends are expected to continue. The increasing demand for fish highlights the need for the sustainable management of aquatic resources; 87.3% of world fish stocks are classed as overexploited, depleted or recovering: a number which continues to increase [1], with 29.9% of stocks classed as overexploited and unlikely to meet the targets of the Johannesburg Plan of Implementation to restore them to a level that can produce maximum sustainable yield by 2015 [3].

A major threat for the sustainable management of these valuable resources is Illegal, Unreported and Unregulated (IUU) fishing. Current estimates suggest that globally up to 25% of fisheries catches fall within IUU practices [4]–[6], identifying it as the single largest threat to achieving sustainability. Both the FAO [7] and the European Union [8] have placed increasing emphasis on the use of trade measures to prevent IUU-sourced fish and fish products from entering international trade. One component of this increased regulation has required the inclusion of binomial species nomenclature on catch labels throughout the distribution chain [9].

In addition to top down pressure for improved labelling and traceability of fish products, many consumers are increasingly aware of nutritional and environmental issues regarding fisheries, leading to shifts in attitude regarding acceptable species, catch location and catch methods [10]. In parallel, due to globalization of the industry, consumers are encountering an increasing number of fish species and/or an escalation in common names applied to the same species. Such drivers have led to a greater demand for informative labelling, including the use of ‘eco-labelling’. Although labelling to provide additional ecological information about a product is often voluntary, the FAO recognised that it could contribute to improved fisheries management and convened a Technical Consultation in 1998, which resulted in their Guidelines for the Eco-labelling of Fish and Fishery Products from Marine Capture Fisheries [11]. Informative labelling is particularly important for processed items because any recognizable external morphological features are typically removed, leaving consumers reliant on product labelling for content information. However, it has been argued that any such labelling scheme, whether voluntary or legislated, requires policing in order to prevent misuse and fraud [12].The mislabelling of a fish product may be unintentional if, for example, species that are morphologically similar are caught together, such as in many tropical or coral reef fisheries [13]–[17]. Alternatively, mislabelling may not be accidental, such as where product substitutions are from species that do not occur in the same ocean [18]–[20], or for lesser value species [21], [22]. However, whether intentional or not, the outcome can be serious for management and sustainability targets. In addition to the direct impacts of depletion from IUU fishing, substitutions and misidentification that occur before fish are landed will inflate the inaccuracies in catch and forecast statistics.

Several recent studies of mislabelling have been undertaken in Europe [19], [22], [23], yielding rates of mislabelling of up to 32% [19]. Most mislabelled products have originated from small-scale retailers and convenience food outlets (e.g. fish and chip shops) but the major supermarkets have not hitherto been thoroughly investigated. Supermarket chains account for 72% of the total fish retail market in the UK (excluding canned products) [24]. If comparable rates of mislabelling occur in supermarket products it is thereby likely to have a substantial impact on efforts to manage the respective fisheries sustainably. It is therefore necessary to establish to what extent mislabelling of fish products occurs in the major retailers of the fish food supply chain, which is addressed in this study.

The current study uses two DNA-based methods to identify the species of origin for 386 samples collected from major supermarket chains around the UK. Species-specific real-time PCR probes [25] for cod (*Gadus morhua*), and haddock (*Melanogrammus aeglefinus*) were used to provide a highly sensitive test for the major species of white fish sold in British supermarkets. Additionally, DNA barcoding [26] using fish-specific COI primers [27] was employed. The COI mitochondrial gene has been validated for forensic species identification [28] to determine its reproducibility and limitations by testing its ability to provide accurate results under a variety of conditions. To our knowledge, the current findings represent the first large-scale assessment of fish product authentication across major UK supermarket retailers.

## Materials and Methods

### Sample collection

386 samples of processed white fish, ranging from fillets to fish fingers and fish cakes, were collected from six leading supermarket chains, at multiple locations across England, Scotland and Wales (Table S1in File S1). Approximately 20 mg of tissue was taken from the centre of each product to ensure minimal DNA damage from production, processing, or contamination. These were placed into numbered tubes filled with 96% ethanol. Sample details including the place and date of purchase, species designation, and eco-labelling were entered into a database linked to photographs of the packaging. Sample identities were not disclosed until completion of molecular genetic analyses, when molecular and sample IDs were cross-referenced.

### Molecular methods

DNA was extracted with the E-Z 96 Tissue DNA kit (Omega-biotek), then quantified with a Nanodrop 1000 (Thermo Scientific), and standardised to either 5 ng/µL or 2 ng/µL depending on original concentration. Real-time PCR assays were carried out on all samples on an Applied Biosystems 7700 real-time sequence detection system. The 25 µL reactions contained 200 nM of each of the two species specific probes (see Table 1), 300 nM of the GAD-F and GAD-R primers (Taylor *et al*. 2002), 9.163 µl 2X Taqman Universal PCR Master Mix (UNG+ROX and passive reference) (Applied Biosystems), 15 ng of DNA, and (depending on DNA concentration) either 10.417 or 6.917 µL PCR grade H_2_O (Sigma). Reactions were run in optical 96-well reaction plates using optical adhesive covers (Applied Biosystems). Plates were analysed under real-time conditions on two dye layers with eight ‘no template controls’ (NTCs) per 96-well plate, and 2 positive controls for each of the two target species. The assay was run using the default cycling conditions [25].

**Table 1:** list of all primers used.

	Sequence 5′-3′	Reporter	Quencher
COD P	CTTTTTACCTCTAAATGTGGGAGG	-	-
HAD P	CTTTCTTCCTTTAAACGTTGGAGG	-	-
GAD-F	GCAATCGAGTYGTATCYCTWCAAGGAT	FAM	Non-fluorescent
GAD-R	CACAAATGRGCYCCTCTWCTTGC	TET	Non-fluorescent
FishF1	TCAACCAACCACAAAGACATTGGCAC	-	-
FishR2	ACTTCAGGGTGACCGAAGAATCAGAA	-	-

COD P, HAD P, GAD-F, and GAD-R were used in the real-time-PCR, and FishF1 and FISHR2 were used for the sequencing PCRs.

In addition to the real-time PCR, all samples were sequenced for approximately 655 bp from the 5′ region of the COI gene from mitochondrial DNA using primers developed by Ward [27]. Tests were run with all combinations of the four available primers, but the combination of FishF1/FishR2 produced consistently good PCR products in the species tested, and was therefore used throughout (see Table 1). PCRs were carried out in 30 µL reactions containing 15 µL of 2 x PCR Mastermix (containing 0.75 U of *Taq* polymerase (buffered at pH 8.5), 400 µM each dNTP, 3 mM MgCl_2_ (Promega)), 9 µL PCR grade H_2_O (Sigma), 15 pmol each primer, and 3.0 µL of DNA template. The PCRs consisted of a denaturation step of 2 min at 95°C followed by 35 cycles of 30 seconds at 94°C, 30 seconds at 54°C, and 1 min at 72°C, followed by a final extension of 10 min at 72°C and then held at 4°C. PCR products were visualized on 1.2% agarose gels. If a single clear band was produced, PCR products were sent to GATC (Germany, http://www.gatc-biotech.com) for sequencing. DNA from 48 samples was re-extracted as independent replicates of real-time PCR and sequencing, including all samples where molecular data contradicted species designations, and an additional randomly chosen 33 samples to test repeatability of DNA-based species ID.

### Species identification

#### Real-time PCR

The results were analysed using the Sequence Detection Software version 1.71 (Applied Biosystems). The ΔRn values for each cycle and dye layer were then exported to MS Excel and additional manual processing was carried out. First, the mean and standard deviation of the endpoint (PCR cycle 40) ΔRn values of the NTCs were calculated for each dye layer. z*M-values (z*M = M+(3.89xSD)+C) were then calculated where M = mean of the NTC ΔRn, SD is the standard deviation of the NTC ΔRn and 3.89 is the one tailed *Z*-value for the 99.999% confidence interval, C is a constant (0.3) introduced to overcome the slight increase in fluorescence of samples above the NTC fluorescence due to spectral bleeding between dye layers. Samples which had ΔRn values larger than the value of z*M were considered to have a fluorescence significantly greater than the NTCs, and therefore to be positive reactions.

#### COI sequencing

Successfully sequenced COI amplicons were manually checked and edited to remove ambiguous base calling in BioEdit (Ibis Biosciences). Sequences were tested against the Barcode of Life database (BOLD) [29]. In addition, reference sequences for all species genetically identified and all species indicated on sample packaging, were downloaded from BOLD and aligned with the sample sequences in Clustal X [30], the Neighbour-joining tree was constructed in MEGA5 [31] with 1000 bootstrap replicates.

## Results

For consistency, all samples are referred to by the labelled species unless otherwise stated. Of 386 samples, 371 (97.4%) produced DNA of sufficient quality for further analysis. Label designations indicated primarily cod (179), haddock (155) and pollock (32).

**Real-time-PCR.** All samples labelled as hake or Alaskan pollack showed negative results for probes designed to identify cod and haddock. The sample labelled as whiting was positive for cod. For the samples labelled as haddock (155), the haddock probe was positive in 134 samples (86.5%), while the cod probe gave a positive result for 6 samples, both probes were amplified in 7 samples (inconclusive result) and neither were amplified in 8 samples (negative). All cod labelled as originating from the Pacific were negative for both the cod and haddock probes. Out of the Atlantic cod samples (57), the cod specific probe amplified in 47 samples (82.5%), both probes were positive in 3 samples (inconclusive result) and neither in 7 samples (negative). For the cod samples which did not indicate a catch location (102), the cod specific probe was positive in 80 samples, the haddock specific probe was positive in 2 samples, both amplified in 8 samples (inconclusive result) and neither in 12 samples (negative). Real-time-PCR results are presented in Table 2.

**Table 2:** Summary of all mislabelled samples.

Identification Code	Species reported (type)	Area of Catch	real-time PCR	First sequence identity	Second sequence identity
1415	Cod (breaded fillet)	Atlantic	Negative	*Gadus macrocephalus*	*Gadus macrocephalus*
1426	Cod (breaded fillet)	Atlantic	Negative	*Gadus macrocephalus*	*Gadus macrocephalus*
1446	Cod (breaded fillet)	Atlantic	Negative	*Gadus macrocephalus*	*Gadus macrocephalus*
1747	Cod (precooked meal)	Atlantic	Negative	*Gadus macrocephalus*	*Gadus macrocephalus*
1889	Cod (precooked meal)	Atlantic	Negative	*Gadus macrocephalus*	*Gadus macrocephalus*
1975	Cod (breaded fillet)	Atlantic	Negative	*Gadus macrocephalus*	*Gadus macrocephalus*
1886	Cod (fish cakes)	NA	Inconclusive	*Gadus morhua*	*Melanogrammus aeglefinus*
1765	Cod (fish cakes)	NA	*Melanogrammus aeglefinus*	*Melanogrammus aeglefinus*	*Melanogrammus aeglefinus*
1892	Cod (fish fingers)	NA	*Gadus morhua*	*Gadus chalcogrammus*	*Gadus morhua*
1470	Haddock (precooked meal)	Atlantic	*Gadus morhua*	*Gadus morhua*	*Gadus morhua*
1812	Haddock (fish cakes)	Atlantic	*Gadus morhua*	*Gadus morhua*	*Gadus morhua*
1888	Haddock (precooked meal)	Atlantic	*Gadus morhua*	*Gadus morhua*	*Gadus morhua*
1977	Haddock (breaded fillet)	Atlantic	*Gadus morhua*	*Gadus morhua*	*Gadus morhua*
1989	Haddock (precooked meal)	Atlantic	*Gadus morhua*	*Gadus morhua*	*Gadus morhua*
1868	Haddock (precooked meal)	Atlantic	*Gadus morhua*	*Gadus morhua*	*Gadus morhua*
1851	Haddock (precooked meal)	Atlantic	Negative	*Gadus macrocephalus*	*Gadus macrocephalus*
1452	Haddock (fish cakes)	Atlantic	Inconclusive	*Gadus morhua*	*Melanogrammus aeglefinus*
1847	Haddock (fish cakes)	Atlantic	Inconclusive	*Gadus morhua*	*Melanogrammus aeglefinus*
1763	Alaskan Pollack (fish cakes)	Pacific	Negative	*Pangasius hypophthalamus*	*Gadus chalcogrammus*
1813	Hake *(M. capensis)* (breaded fillet)	NA	Negative	*Merluccius paradoxus*	*Merluccius paradoxus*
1848	Whiting (precooked meal)	NA	Inconclusive	*Micromesistius poutassou*	*Micromesistius poutassou*

NA: Not available from packaging. Negative: neither of the real-time PCR probes amplified. Inconclusive: both real-time PCR probes amplified. First and second sequence identities are the result of independent DNA extractions and sequencing (see methods for details).

**COI sequencing.** All sequence data has been submitted to NCBI, under accession numbers KJ614671 to KJ615069 (Table S2 in File S1). 48 samples have two sequences listed as these samples were re-extracted as independent replicates to ensure the repeatability of the methods.

The majority of sequences were identified with a sequence identity greater than 99.5% in the BOLD database, with sequences from two samples falling below this threshold. Additionally, two samples could not be matched unambiguously due to 100% sequence identity at COI at the taxon-pairs involved. The sequence data matched with *Gadus chalcogramma/G. finnmarchica* (Alaskan and Norwegian Pollock respectively; previously *Theragra* sp.), or *Gadus macrocephalus* and *Gadus ogac* (Pacific and Greenland cod respectively): these are both instances where the (sub-) species designation is debatable (see Discussion).

Of 179 samples labelled as cod, 57 were specified as Atlantic cod (*Gadus morhua*) and 20 as Pacific cod (*Gadus macrocephalus*), while for the remainder (102) there was no specification for either the species or catch area. In total, 9 (5.03%) of these cod samples were not verified as cod by DNA data, including 1 (0.56%) identified as *Melanogrammus aeglefinus* (haddock), and 2 (1.11%) highly processed samples that were found to have a mixed species composition (see Table 2: #1892; *G. morhua/G. chalcogramma* and #1886; *G. morhua/M. aeglefinus*). From the 57 samples labelled specifically as Atlantic cod, 51 had congruent label and DNA-based designations, while 6 (10.5%) were genetically identified as Pacific cod (*G. macrocephalus*).

155 samples were labelled as haddock (*M. aeglefinus*). Of these, 146 generated a molecular ID in agreement with labelling (5.81% mislabelled), with 6 (3.87%) identified as *G. morhua* (Atlantic cod), 1 (0.65%) as *G. macrocephalus* (Pacific cod) and 2 (1.29%) exhibited a mixed species composition (see Table 2: #1452 and #1847; *G. morhua/M. aeglefinus*).

**Figure 1:**
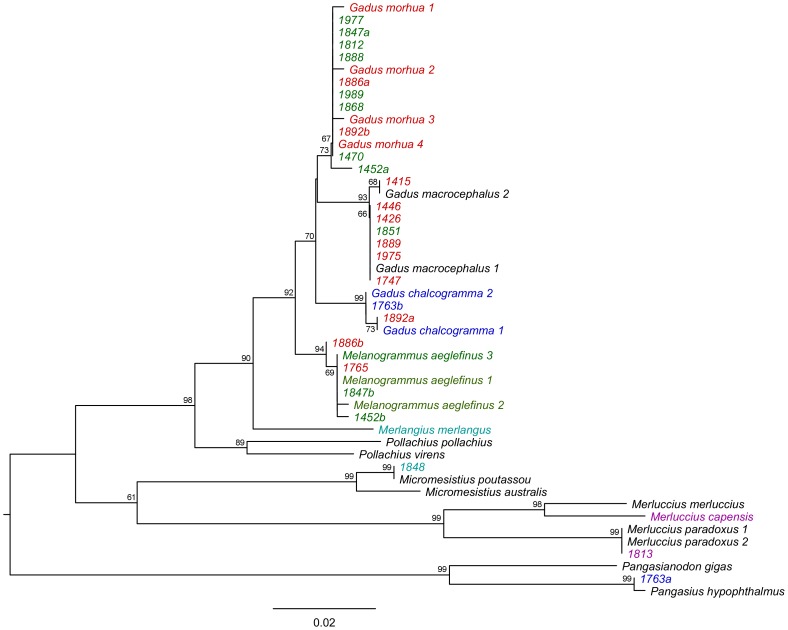
Neighbour-joining tree showing all mislabelled samples together with representative reference sequences taken from BOLD. Reference sequences are colour coded according to species and samples tested are colour coded according to the species stated on the packaging. Samples that have two sequences are labelled a and b.

In addition, one of the four hake (labelled as *Merluccius capensis*) samples was identified as *Merluccius paradoxus* (cape hake), one whiting (*Merlangius merlangus*) sample was identified as *Micromesistius poutassou* (blue whiting), and one Alaskan Pollack was also found to contain the Vietnamese catfish *Pangasius hypophthalmus*. Overall, our survey indicated a rate of mislabelling of 5.66%. All samples and results are presented in Tables S1 and S2 in File S1, with detailed results of the mislabelled samples in Table 2 and Table S3 in File S1. Sequence similarity with all reference samples is demonstrated in Figure 1, and the details of the reference sequences used are in Table S4 in File S1.

## Discussion

Our study represents, to our knowledge, the largest published survey to date of mislabelling within the fish products sold by UK supermarkets. Samples were taken of products from leading brands and supermarket “own brands” from 6 major supermarket chains across the UK. Previous studies have examined the food retail sector and found high rates of mislabelling, particularly in restaurants and fast-food outlets [22], [32]. Within our study of supermarket-sourced samples the overall inconsistency between product label and genetic species identification was 5.66%. This is considerably lower than observed in other sectors: 25% within mixed sectors [22]; 25% within markets and restaurants [32]; 32% within fishmongers [19]. Nevertheless, if our data are representative of overall trends, with over 4 billion fish products consumed (C. Roberts, unpublished data) the incidence of mislabelling could exceed 200 million products annually in the UK alone. This level of misinformation raises considerable concern in terms of consumer information and protection. It also presents substantial challenges for the sustainable management of the respective fisheries.

Genetic identification of products was carried out with species specific real-time PCR, and by matching sample COI sequences with those of known species in the BOLD database with high (≥99.5%) sequence identity [33]. Such independent testing yields a high degree of certainty to the identifications, as more than 98% of species pairs have shown greater than 2% COI sequence divergence [34]. The BOLD database was used in preference to the nucleotide sequence database in GenBank (www.ncbi.nlm.nih.gov/), to ensure that the queried sequences were matched to taxonomically-validated specimens. Of all the sequences submitted, only two returned a match with less than 99.5% identity. Both of these were from highly processed samples (one labelled as cod, the other as haddock), and also returned inconclusive results for the real-time-PCR (both cod and haddock probes amplified). Both sequences were genetically identified as *M. aeglefinus* (haddock), although with relatively low sequence similarity (99.49% and 98.6%). For both of these sequences, the next closest match was *G. morhua*, rather than the next closest relative of haddock, *Merlangius merlangus* (see Figure 1), supporting the conclusion that the DNA amplified was a mix of more than one species, and therefore that these products had a mixed species composition.

Ambiguous results occurred when a sample matched with both Alaskan and Norwegian pollock (*Gadus ( = Theragra) chalcogramma* and *G. finnmarchica*, respectively), or with Pacific and Greenland cod (*Gadus macrocephalus* and *G. ogac*, respectively), because congeners have 100% sequence identity at COI. However, in the case of Pacific and Greenland cod, catches of *G. ogac* are thought to be extremely low, and currently only of local importance. The total reported catch for this stock from 2009–2011 was 586 metric tons [35], while for the same three years, the total reported catch for *G. macrocephalus* was 1,165,420 metric tons. Greenland cod is also no longer considered a separate species, but is now classed as a subspecies of Pacific cod, *G. macrocephalus* [36], [37]. In the case of the Pollack species, *G. finnmarchica* was identified from a few samples from the northern tip of Norway [38] and recent molecular evidence has shown it to be indistinct from the Alaskan Pollock (*G. chalcogramma*) [39]–[41].

From all samples labelled as Atlantic cod, the majority of those found to be mislabelled were genetically identified as Pacific cod. This category of mislabelling could not originate at the pre-landing stage; as is evident from their common names; these species are harvested from different oceans. The implication, therefore, is that intentional mislabelling has occurred at a later stage in the supply chain. The incentive could be to supply products that mirror the preferences of the buying public, and so presumably fetch a higher price. This class of mislabelling may have little direct impact on the Atlantic cod stocks but it may influence efforts to sustainably manage stocks of Pacific cod. More importantly perhaps for this particular case of mislabelling is the issue of consumer misinformation and protection as it indicates that at some point in the supply chain there appears to be either negligence or a wilfully fraudulent attempt to provide inaccurate product information. Such instances erode consumer confidence and can undermine trust in product labelling, including any associated eco-labels.

Samples labelled as *M. aeglefinus* (haddock) show a different pattern of mislabelling. The majority of mislabelled products were identified as *G. morhua* (Atlantic cod). Haddock and Atlantic cod are frequently caught together in a mixed fishery and have similar market values, with cod slightly more valuable on average. As a result there is minimal direct benefit to intermediaries in the production chain to encourage such mislabelling. Alternatively, it has been suggested that such mislabelling may arise by an accidental consequence of the mixed fishery [23]. However, while we accept such possibility, mislabelling undeniably benefits the primary producer. Mislabelling *G. morhua* (Atlantic cod) as *M. aeglefinus* (haddock) enables fishermen to land undersized or over quota Atlantic cod and so profit from fish that should currently be discarded. Irrespective of the underlying cause, if the mislabelling occurs before the fish are landed (for example, if filleted and frozen at sea), such IUU activities will likely exceed catch quotas (TAQ) for a major North Atlantic fishery. The rate of mislabelling (3.87%) is comparatively low compared to other recent studies [22]. However, if we extrapolate such incidence to the TAQ for 2011, it represents an additional 2188 tonnes of Atlantic cod (or an excess 2.9% of the Atlantic cod TAQ for 2011) being landed and recorded as haddock.

In addition to the mislabelling of cod and haddock presented here, other mislabelling instances were found. One highly processed (fish cake) sample labelled as containing Alaskan Pollack (*G. chalcogramma*) was found to also contain *Pangasius hypophthalmus. P. hypophthalmus*, or Vietnamese catfish, is a freshwater species from Southeast Asia, legally described in the UK as Basa, Panga(s), *Pangasius*, River cobbler or any of these combined with ‘catfish’ [42]. Without performing a quantitative test for the presence of *P. hypophthalmus*, we were unable to estimate the relative quantities of the 2 species in this product (made of minced fish). It was therefore not possible to determine whether this reflected inadvertent contamination through inadequate cleaning of the production line between products, or deliberate substitution of a cheaper product. In either case it is unlikely to significantly affect catch data or to contribute to IUU. However, this accidental or fraudulent behaviour is a serious issue for consumer misinformation and trust, given the concerns over potentially increased contaminant levels in *Pangasius* species (such as mercury) [43], which may result in avoidance by some consumer groups.

Four hake samples were tested and one, labelled as *M. capensis*, was identified as *M. paradoxus* (25% mislabelled, although the low sample size requires caution). Historically, hake has been assessed as a single species, as separation of catches has not always been possible [44], [45]. However species-specific assessments are now being conducted. The shallow water *M. capensis* stock is above sustainable levels, with catches below maximum sustainable levels and is certified by the Marine Stewardship Council (MSC). The deep-water *M. paradoxus* stock is below precautionary levels, and a rebuilding plan is in place [46]. The mislabelling of this species, whether intentional or not, at a rate even well below that observed here is a cause for serious concern, as such a practice would compromise restoration of *M. paradoxus* to sustainable levels.

One noteworthy pattern to emerge is the variation in amount of mislabelling found among the different levels of processing: within the fresh/frozen fillets (n = 84) no mislabelling was identified; in battered/breaded fillets (n = 84), fish fingers (n = 31), and pre-cooked meals (n = 128), the respective mislabelling rates were 7.14%, 6.45% and 5.47% respectively. In fishcakes (n = 44), which are composed of minced fish, mislabelling rates of 13.6% were identified. However, these data are insufficient to identify where in this production chain, pre- or post-landing, yield higher rates of illegal activity. Targeted sampling at discrete stages across the supply chain is required: from on-board during sample catch to final retailer outlet. Alternatively it may be an inadvertent consequence of the particular processing activity, such as inadequate cleaning of processing machinery. Huxley-Jones et al. [23] found lower levels of mislabelling in processed products, such as fish fingers, than filleted products, and suggested that this may be due to greater economic gains associated with the mislabelling of fillets. In contrast, our study included more diverse forms of processing (from fresh fillets through fish fingers and precooked meals to fish cakes consisting of minced fish), and has demonstrated a clear pattern of mislabelling, from zero in unprocessed fish fillets to the highest levels of mislabelling in the most highly-processed category.

The main trends highlighted here have been the substitution of *G. morhua* (Atlantic cod) with *G. macrocephalus* (Pacific cod) in primarily filleted products, and the substitution of *M. aeglefinus* (haddock) with *G. morhua* (Atlantic cod) in precooked meals and fish cakes. Both aspects of mislabelling have a detrimental effect on *G. morhua*: substitution with *G. microcephalus* creates an erroneous impression of the abundance of the former, undermining work carried out by seafood awareness campaigns such as Seafood Watch and the Marine Stewardship Council, to educate consumers and provide tools for informed purchasing decisions. However, cod is one of the species for which there are now sufficient genomic resources to move beyond species identification and allow traceability to population level [47]. Testing by regulatory and certifying bodies would improve consumer confidence in products that are proven to fulfil claims of having been sourced from sustainably harvested stocks. In addition, as suggested here, if the substitution of *M. aeglefinus* with *G. morhua* is occurring at sea, the implications of such IUU activity would compromise the recovery of these heavily exploited species.

Previous studies have reported relatively high rates of mislabelling of seafood products globally [13], [48], in Europe [19], [22], [49], and South Africa [21], [50]. However, many studies have focused on smaller convenience food outlets and/or restaurants. Actions such as increasing media attention, the importance of consumer confidence in the fisheries sector and revised EU legislation [51], [52] will collectively highlight and tackle mislabelling practices. Nevertheless, only genetic testing across the supply chain can assess the scale and likely key stages of highest risk. It also appears increasingly likely that such practices are more frequent at the more highly processed end of the market, where opportunities for detection and/or levels of discrimination are reduced. As witnessed recently in the wake of the horsemeat scandal across Europe, the complexities of the modern food production chain demand close scrutiny at all stages to ensure authenticity and compliance. A forensic framework of genetic testing using validated reference databases [47], [53]–[55] is expected to provide an increasingly effective approach for detection, prosecution and ultimate deterrence of illegal activity. Such actions are likely not only to protect policy compliant end-users and the wider fishing industry but importantly also enhance prospects for achieving sustainability of exploited marine resources.

## Supporting Information

File S1 **Supporting Information.** **Table S1.** Summary of sampling effort for samples which produced a DNA of sufficient quality for testing. Samples are recorded by reported species and are split by supermarket (own brand/other brand items). **Table S2.** Genetic analyses for all samples. The sample identification number, product labelling (reported species and catch location), processing level* and results from the real time PCR and COI sequencing are given for each sample. Second sequence identity is the result of new DNA extraction and sequencing for mislabelled, ambiguous, and control samples. Genbank accession numbers are provided for each sequence. * classification of processing level (1: fresh or frozen fillets, 2: battered or breaded fillets, 3: fish fingers, 4: pre-cooked meals, 5: fishcakes). **Table S3.** Additional data for the mislabelled samples. Query sample details, including species labelled on packaging, COI sequence and the Genbank accession number are given. Reference species is the closest sequence match in the BOLD database, together with the catch location, BOLD ID number and Genbank accession number of the reference sample. Sequence similarity (% identity) between sample and reference is shown (* indicates sequence matches lower than 99.5%). Sequence similarity to the next closest match is also shown. **Table S4.** BOLD and Genbank identifiers for the reference sequences used in Figure 1.
(XLSX)
